# Calcification by Reef-Building Sclerobionts

**DOI:** 10.1371/journal.pone.0060010

**Published:** 2013-03-28

**Authors:** Jennie Mallela

**Affiliations:** 1 Research School of Biology^,^ The Australian National University, Canberra, Australian Capital Territory, Australia; 2 Research School of Earth Sciences, The Australian National University, Canberra, Australian Capital Territory, Australia; 3 The Department of Life Sciences, University of the West Indies, St Augustine, Trinidad and Tobago, West Indies; Leibniz Center for Tropical Marine Ecology, Germany

## Abstract

It is widely accepted that deteriorating water quality associated with increased sediment stress has reduced calcification rates on coral reefs. However, there is limited information regarding the growth and development of reef building organisms, aside from the corals themselves. This study investigated encruster calcification on five fore-reefs in Tobago subjected to a range of sedimentation rates (1.2 to 15.9 mg cm^−2^ d^−1^). Experimental substrates were used to assess rates of calcification in sclerobionts (e.g. crustose coralline algae, bryozoans and barnacles) across key reef microhabitats: cryptic (low-light), exposed (open-horizontal) and vertical topographic settings. Sedimentation negatively impacted calcification by photosynthesising crustose coralline algae in exposed microhabitats and encrusting foram cover (%) in exposed and cryptic substrates. Heterotrophs were not affected by sedimentation. Fore-reef, turbid water encruster assemblages calcified at a mean rate of 757 (SD ±317) g m^−2^ y^−1^. Different microhabitats were characterised by distinct calcareous encruster assemblages with different rates of calcification. Taxa with rapid lateral growth dominated areal cover but were not responsible for the majority of CaCO_3_ production. Cryptobiont assemblages were composed of a suite of calcifying taxa which included sciaphilic cheilostome bryozoans and suspension feeding barnacles. These calcified at mean rates of 20.1 (SD ±27) and 4.0 (SD ±3.6) g m^−2^ y^−1^ respectively. Encruster cover (%) on exposed and vertical substrates was dominated by crustose coralline algae which calcified at rates of 105.3 (SD ±67.7) g m^−2^ y^−1^ and 56.3 (SD ±8.3) g m^−2^ y^−1^ respectively. Globally, encrusting organisms contribute significant amounts of carbonate to the reef framework. These results provide experimental evidence that calcification rates, and the importance of different encrusting organisms, vary significantly according to topography and sediment impacts. These findings also highlight the need for caution when modelling reef framework accretion and interpreting results which extrapolate information from limited data.

## Introduction

Worldwide, reef-building organisms are under increasing sediment stress from natural (e.g. storm and catchment runoff) and anthropogenic disturbances (e.g. land use change, dredging) which have resulted in the subsequent decline in near-shore water quality [1], [2]. Unfortunately, we know very little about how these disturbances impact secondary reef development. A coral reef is the product of net accumulation of calcium carbonate (CaCO_3_) which is laid down by numerous carbonate-secreting organisms. These include primary reef building corals and secondary reef builders, e.g. crustose coralline algae, bryozoans, foraminifera and serpulid worms [3]–[5]. Scleractinian corals are typically the major reef builders with carbonate production ranging from <1 kg m^−2^ y^−1^ on degraded reefs to 14.3 kg m^−2^ y^−1^ on healthy, clear water reef sites [6], [7]. Fore-reef encruster carbonate production is also thought to contribute significantly to the reef framework with the limited data available reporting values ranging from 0.05 for all encrusters to 3.6 kg m^−2^ y^−1^ for CCA [8], [9].

Globally, increased catchment runoff (e.g. sediment and nutrients) threatens reefs by elevating turbidity and sedimentation and causing eutrophication [1]. Numerous studies have documented sediment stress responses in reef-building corals which include: declining calcification; reductions in live coral cover; reduced larval settlement and loss of coral diversity, for reviews see: [10], [11]. Sediment is also thought to negatively impact benthic cover by secondary reef building organisms (e.g. CCA and total encruster cover) [12], [13]. However, there is limited information available on the impacts of sediment on secondary reef building organisms, but see bulk estimates for 'all encrusters' [9], [13].

The rate of calcification associated with reef-building organisms is a key indicator of reef health. However, due to a paucity of information, relatively little is known about rates of calcification for different encrusting, carbonate-secreting sclerobionts, sensu Taylor [14], also referred to as encrusters. Encrusters include, but are not limited to: bryozoans, CCA, foraminifera, and serpulid worms. These organisms promote reef accretion by colonising reef substrates, depositing calcium carbonate, consolidating and stabilising existing framework, inducing larval recruitment from other organisms, enhancing topographic complexity and maintaining wave resistant reef fronts [13], [15]–[17]. As corals typically dominate carbonate production, they have been the primary focus of many reef accretion studies [4], [9], [18], [19]. However, in some reef settings, encrusters can dominate carbonate production, e.g. coralline algal ridges have been documented in shallow, wave impacted, reef crest zones [20], [21].

Most encruster studies have focused on ecology and community development, relying on visual estimates of encruster cover (%), morphological assessments and linear growth rate measurements (e.g. mm y^−1^) [22]–[25]. Calcification (g m^−2^ y^−1^) is the combined result of both linear growth (extension mm y^−1^) and skeletal density (g cm^−3^). Focusing on only one growth parameter can be misleading when assessing reef accretion, as high extension rates do not always correspond with high calcification rates [4]. For example, unusual increases in skeletal extension in corals have been associated with declining water quality [7]. Rapid linear extension combined with reduced skeletal density can result in more porous coral skeletons and a weaker reef framework, which may be more prone to breakage during high energy events, e.g. storms [26]. However, relatively few studies have documented encruster calcification [9], [27], [28], with the majority focusing on coralline algae [29]–[31] or bulk (combined) encruster calcification [13]. As few studies have quantified fine-scale or organism specific rates of calcification for encrusters, prior estimates of reefal carbonate production (varying from local to global scales) have simply extrapolated from a limited number of reef studies, e.g. [27], [32]. Interestingly, initial findings have highlighted significant differences in carbonate production by encrusting organisms within different reef topographic settings [13]. As a result there are potentially flaws in global models that rely on extrapolation from limited data sets to assess how reef accretion will respond to environmental change. Clearly there is a need for fine-scale (e.g. reef habitat and zone), taxon-specific information regarding calcification and reefal carbonate accretion.

In the present study, community composition and annual calcification rates for multiple sclerobionts growing in three distinct micro-habitats were assessed. Microhabitats included: upwards facing, light exposed (e) substrates, downwards facing, cryptic-shaded (c) substrates, and vertical, light-exposed substrates (v). This study assessed: 1) which encruster groups distinguished communities in different microhabitats; 2) encruster calcification on fore-reefs; 3) if sedimentation impacted encruster cover (%); and 4) if calcification rates for CCA, encrusting bryozoans, barnacles (episkeletozoans) and ‘other’ encrusters varied with a) substrate orientation, or b) sediment impacts.

## Materials and Methods

### Ethics Statement

No specific permits were required for the described field studies. The activities for this study were conducted in collaboration with the Tobago House of Assembly.

Tobago’s reefs are unique, representing some of the southern most reefs in the Caribbean. They represent persistent, turbid-water framework accreting reefs [33]–[35]. The reefs are adapted to seasonal pulses of turbid, nutrient rich river runoff from regional and local sources [36]. The amount of total suspended sediment on Tobago’s reefs is often <10 mg L^−1^, but following runoff and high energy events can exceed 200 mg L^−1^ (Table 1). In recent decades, the reefs have been subjected to additional climate and human induced stressors which include declining water quality [36]–[38], large scale temperature-induced bleaching [2], [39], subsequent coral disease [40], and overfishing [41]. At the time of this study, Tobago’s reefs were considered to be threatened and at high risk from the combined effect of these stressors [1]. Despite this, and their importance to the provision of ecosystem goods and services [42], relatively little is known about their growth and development.

**Table 1:** Mean (±SD) water quality parameters (mg L^−1^) for sites on the north coast of Tobago: total suspended solids [44], phosphate, nitrate, ammonia and nitrite.

Site	TSS	*± SD*	Phosphate	*± SD*	Nitrate	*± SD*	Ammonia	*± SD*	Nitrite	*± SD*
Kariwak	84.00	*125.80*	0.02	*0.02*	0.05	*0.03*	0.03	*0.03*	0.01	*0.01*
Buccoo	16.10	*8.30*	0.05	*0.02*	0.07	*0.04*	0.02	*0.01*	0.02	*0.03*
Mt Irvine	27.33	*16.17*	0.09	*0.12*	0.03	*0.01*	0.05	*0.04*	0.01	*0.01*
Culloden	13.80	*7.50*	0.03	*0.14*	0.06	*0.04*	0.03	*0.01*	0.01	*0.00*
LEB	16.85	*9.59*	0.07	*0.06*	0.07	*0.03*	0.02	*0.02*	0.02	*0.02*
Sisters-control	14.25	*8.18*	0.10	*0.08*	0.06	*0.04*	0.02	*0.02*	0.01	*0.01*
Kruskal-Wallis (H)					3.011				0.298	
*p*					*0.698*				*0.998*	
ANOVA (F_5, 17_)	*1.055*		*1.188*				*1.020*			
*p*	*0.419*		*0.356*				*0.437*			

Fore-reef sites sampled at a depth of 10 m in March, June, August and November, 2007 (n = 4). Unpublished data provided by IWCAM [43]. Methods used include: gravimetric determination for TSS - United States Environmental Protection Agency SMEWW 2540 d; phosphate – Hach 8048, nitrate – Hach 8192; ammonia- Hach 8038; nitrite – Hach 8507. Detection limits (mg L^−1^) were: TSS 1.0; phosphate, ammonia and nitrate 0.01; nitrite 0.001. Note: water quality between sites was not significantly different (*p*>0.05).

### Site Descriptions and Water Quality

**Figure 1:**
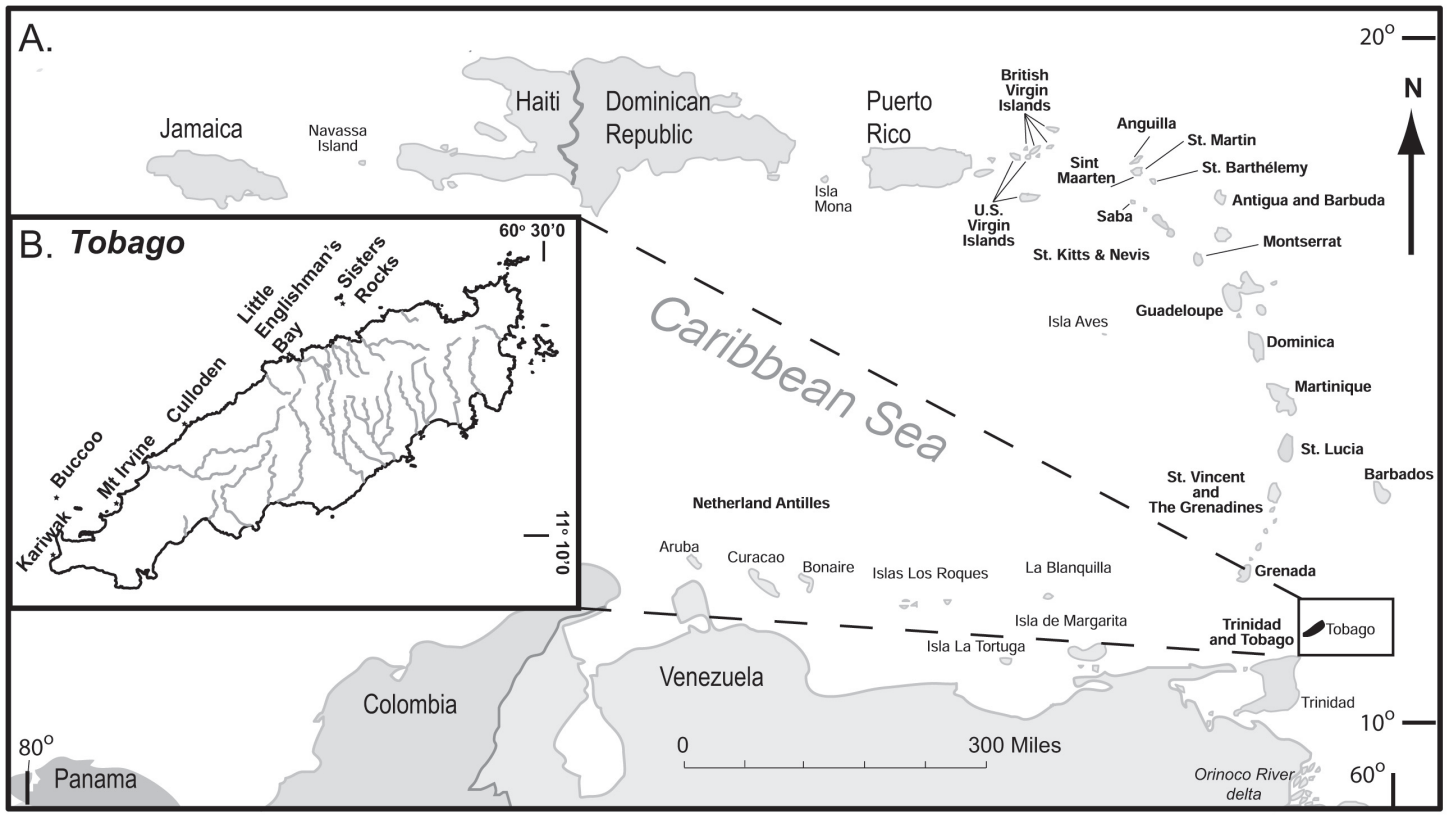
Location of study sites: A. map of the southern Caribbean and B. the location of settlement plates at five reef sites on Tobago’s northern coast and the control site, Sisters Rocks.

**Figure 2:**
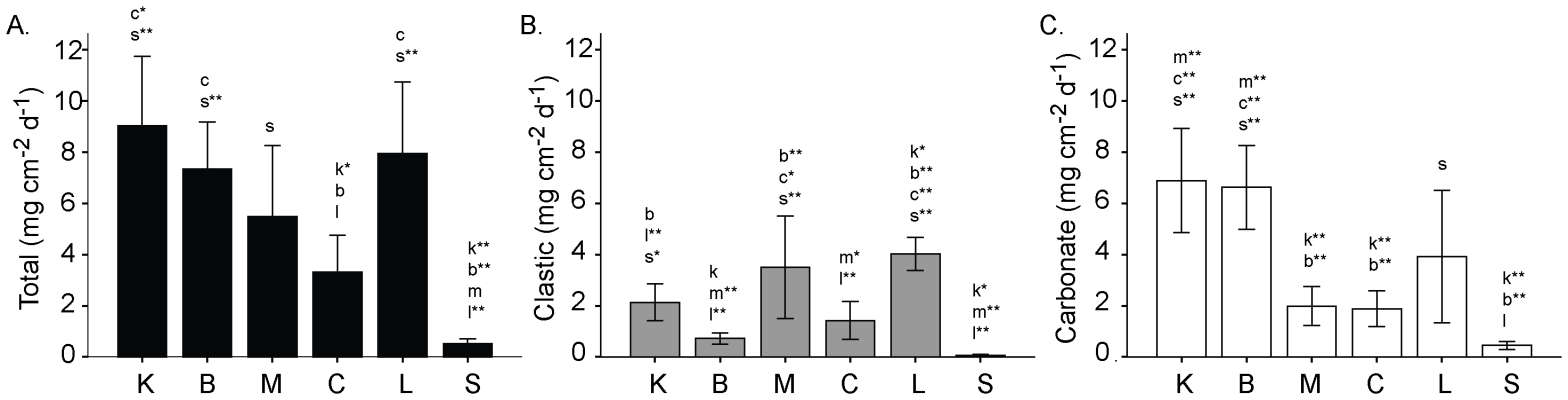
Mean (±95% CI) sedimentation rates for: A. total sedimentation; B. clastic sediment; C. carbonate sediment. Lowercase letters above bars indicate significantly different sites (*p*<0.05, one-way ANOVA with Tukey HSD, n  = 6, *p<0.01, **p<0.001). Lower case letters conform to the first letter in the name of each site.

Six gently sloping, fore-reef sites were selected along Tobago’s north-west coastline. Suitable sites for settlement plates were identified at Buccoo Reef (B), Culloden (C), Kariwak (K), Little Englishman’s Bay (LEB; L), and Mt Irvine (M) with a control water quality site at Sisters Rocks (S) (Figure 1). All sites were in close proximity to the shoreline with the exception of the control site at Sisters Rocks located 3 km west of Tobago. The control, Sisters Rocks, was not subject to direct land based sediment runoff from Tobago, but had similar marine water quality characteristics to the other sites (Table 1). Site specific water quality data (total suspended sediments and nutrients) were sampled four times in 2007 by the Integrating Watershed and Coastal Areas Management Program, Tobago [35], [43]. All sites were well flushed by local currents with marine water quality typical for this stretch of coastline: intermediate-high suspended sediment [44] and nutrient levels (e.g. TSS >10 mg L^−1^, phosphate ≥0.02 mg L^−1^; Table 1, Figure 2).

Sediment impacts differed between sites with mean sedimentation rates ranging from low to moderate [35] (summarised in Figure 2). To determine terrestrial runoff impacts, the rate of clastic (non-carbonate) sedimentation was measured, detailed carbonate-clastic methods given in [9]. In brief, sediment traps (n  = 3 site^−1^) were attached to the reef framework 50 cm above the substrate at 10 m water depth and remained in place from February to October 2007. Traps were positioned adjacent to experiments and collected routinely every month or when logistically possible. Sedimentation (mg cm^−2^ d^−1^) rates were estimated for: total sediment, clastic sediment and carbonate sediment. The carbonate and clastic content of each sediment trap was determined using hydrochloric acid digestion to remove calcium carbonate grains. Sedimentation rates are used here as an indicator of sediment stress at each site.

Benthic communities were generally dominated by scleractinians and gorgonians (Table 2). The exception was LEB, which was dominated by macroalgae. This phaseshift followed sediment runoff from road building activities in 2006 [35]. Additional reef site and water quality information is already published [35], [36], [43].

**Table 2:** Site descriptions characterising the reef framework where experimental tiles were deployed.

Site	Live coral cover (%)	Dominant coral species	Macro algae cover (%)	Site characteristics
Kariwak	14	*Montastrea faveolata*	7.5	Upper Fore-reef, 10 m depth, fringing reef, north coast Tobago
Buccoo	22	*Montastrea faveolata*	2.7	Upper Fore-reef, 10 m depth, fringing reef, north coast Tobago
Mt Irvine	21	*Porites furcata*	1.2	Upper Fore-reef, 10 m depth, fringing reef, north coast Tobago
Culloden	10	*Montastrea faveolata*	5.7	Upper Fore-reef, 10 m depth, fringing reef, north coast Tobago
LEB	18	*Montastrea faveolata*	29.5	Upper Fore-reef, 10 m depth, fringing reef, north coast Tobago
Sisters-control	26	*Diploria strigosa*	12.3	Upper Fore-reef, fringing reef off Sisters Rocks, 3 km offshore.

Benthic data summarised from [35]. Sisters is the control site.

### Experimental Design

Ceramic, unglazed, artificial settlement plates (10 cm x 10 cm) were deployed at the five fore-reef locations. Settlement plates were attached to gently sloping reef framework at a depth of 10 m, ca. 30 cm above the reef substrate. Tiles were attached to PVC poles using cable ties and the poles anchored to reef framework using nails and concrete blocks. At each location 66 plates were positioned horizontally in pairs, with half facing upwards and half facing downwards mimicking exposed (e) and shaded/cryptic (c) reef substrates. An additional 66 plates were positioned in pairs hanging vertically (v) mimicking vertical reef topography. Settlement plates were attached to anchored PVC poles using cable ties. A random selection of 6 plates per orientation were retrieved from each of the 5 locations after 12 months of emersion (May 2007 - May 2008). On collection, the 90 plates were individually bagged underwater and subsequently oven dried at 30°C and air-cooled.

In order to assess the contribution of different calcifying organisms to reef accretion across all sites, community composition was first analysed, using the 30 randomly collected plates per orientation (e, c and v). Encruster community composition was analysed by placing a 100 point grid over each tile and identifying the organism underneath each intersection to taxonomic group (e.g. barnacle, bivalves, bryozoan, CCA, forams, serpulids). Prior work has demonstrated that this approach mimics findings on natural reef substrates [13].

Total calcification rates (g m^−2^ y^−1^) were then estimated for: 1) each plate and 2) three taxa: CCA; barnacles; and cheilostome bryozoans. All other encrusters were grouped together as ‘other’. Calcification rates for each settlement plate were determined following methods detailed in [13]. Briefly, plates were immersed for 24 hours in a solution of 5% domestic bleach, rinsed in distilled water and then oven dried at 50°C and air-cooled to a constant mass (m). Soft, non calcareous organisms (e.g. filamentous algae) were removed using a soft brush and care was taken not to dislodge any calcifying organisms. Plates were weighed (m1) and then placed in a 10% hydrochloric acid bath for 24 hours to remove all calcium carbonate from the tile. Plates were rinsed in distilled water, oven dried at 50°C and air-cooled to a constant mass (m2). Control tests were also run on control plates that had been cleared of all biota manually and subjected to the above treatment. Bulk calcification rates (g m^−2^ y^−1^) were then determined for each tile as m1–m2.

After 12 months, calcification rates were determined for three calcifying groups: CCA; barnacles; and cheilostomes. Plates were weighed, and then a known surface area of each organism (mm^2^) removed carefully from each tile using a scalpel blade. A pre-cut template was used to remove a known surface area for CCA and cheilostomes. The calcareous section was then weighed, and the tile reweighed. CCA and cheilostome sections were typically 1 cm^2^, whilst entire barnacles were removed and the mass and surface area calculated for each individual. Bulk calcification by other organisms was then determined using the method outlined above.

### Statistical Analyses

Data were analysed using SPSS 19 statistical software. Data distributions were determined using the Levene statistic for homogeneity of variance and Kolmogorov-Smirnov to test for data distribution. Normally distributed data were compared using one-way ANOVA with *post hoc* testing where appropriate. Non-parametric data were analysed using Mann-Whitney *U* or Kruskal-Wallis tests. Normally distributed data were correlated using Pearsons product-moment correlation (*r*). Relationships between sedimentation rates and calcifiers were assessed using linear regression analysis (R^2^). Data transformation was not required to meet the assumptions of these tests.

Multivariate analyses were carried out using the PRIMER 6 [45] and PERMANOVA statistical packages [46]. Multivariate data were square-root transformed and the Bray-Curtis similarity coefficient employed to construct a similarity matrix for: A. calcareous encruster communities (% cover); and B. calcification by coralline algae, cheilostomes, barnacles and ‘other’ calcareous organisms. PERMANOVA was used to calculate the distance from centroid of the data cloud grouping by: orientation-site. Matrices were subjected to non-metric multidimensional scaling (MDS) ordinations. One-way analyses of similarities (ANOSIM) tests were used to look for differences between micro-habitats (c, v, e) for A. calcareous encruster cover and B. calcification rates by coralline algae, cheilostomes, barnacles and ‘other’ calcareous organisms. The R-statistic was used to ascertain the extent of any significant differences, R-statistic values <0.1 were regarded as negligible [47]. If ANOSIM detected a significant difference among orientations (R >0.1), Similarity Percentages (SIMPER) [47] was used to identify which organisms made the greatest contribution to different microhabitats.

## Results

### Water Quality

IWCAM [43] marine water quality variables for TSS, phosphate, nitrate, ammonia and nitrite did not vary significantly between sites (*p*>0.05; Table 1). However, sedimentation rates (mg cm^−2^ d^−1^) were significantly different between sites for all three parameters: total sedimentation, carbonate sedimentation and clastic sedimentation (*p*<0.001) (Figure 2). Total sedimentation rates (mg cm^−2^ d^−1^) ranged from 0.3 at Sisters Rocks control site to 14.6 at Kariwak. Terrestrial (clastic) sedimentation (mg cm^−2^ d^−1^) was also highly variable ranging from 6.9 at Mt Irvine to <0.01 at Sisters Rocks.

### Community Composition of Calcifying reef Organisms

**Figure 3:**
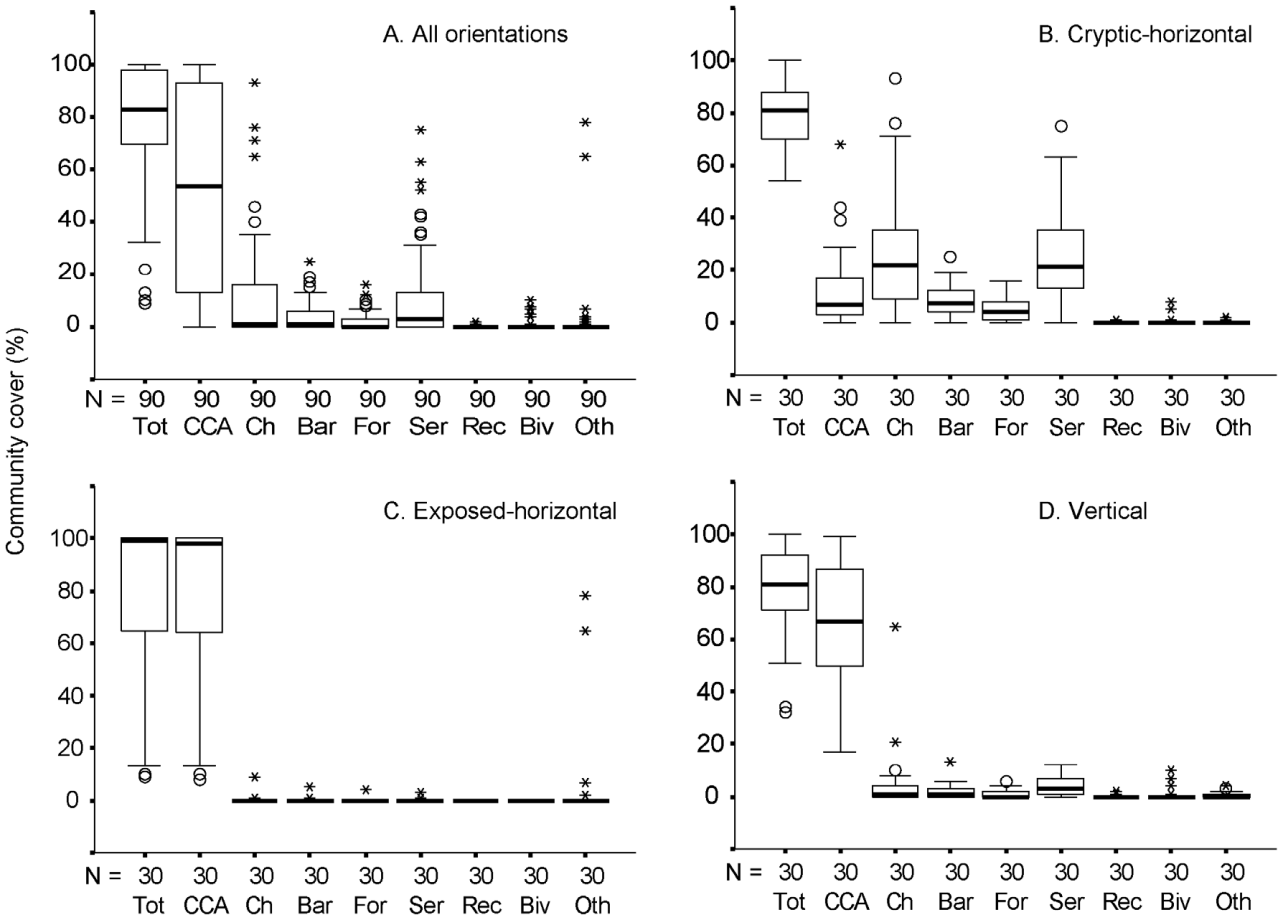
Box whisker plots detailing calcareous encruster cover on: A. all substrates; B. cryptic substrates; C. exposed substrates; and D. vertical substrates. Data presented for calcareous encruster groups: total calcareous cover (Tot); crustose coralline algae (CCA); cheilostome bryozoans (Ch); barnacles (Bar); foraminifera (For); serpulids (Ser); coral recruits (Rec); bivalves (Biv), other calcareous organisms (Oth).

The mean (±SD) total cover (%) of encrusters on all plates was 79 (±21.7), with CCA dominating cover (53.8±36.7), followed by cheilostomes (10.6±19.1), serpulid worms (16.9±17.9), barnacles (6.7±5.6) and forams (2.0±3.3; Figure 3). Total calcareous encruster cover was consistently higher on exposed plates (Mann-Whitney U test: exposed median  = 99%, cryptic median  = 81%, U  = 308.5, *p*  = 0.035, and Mann-Whitney U test: exposed median  = 99%, vertical median  = 81%, U  = 310.5, *p*  = 0.038). No difference in total calcareous cover was observed between the cryptic and vertical plates (*p*  = 0.8).

There was significant variation in the cover (%) of key calcifying organisms between substrate orientations (Figure 3). Significant differences were observed in CCA % cover between orientations (Kruskal-Wallis: *H*  = 48.0, df  = 2, *p*<0.001). Exposed and vertical plates were dominated by CCA (median 98% and 67% respectively). Cryptic plates were characterised by serpulid worms (median 21.5%) and cheilostomes (median 8.8%) and had lower levels of CCA (median 9%).

**Figure 4:**
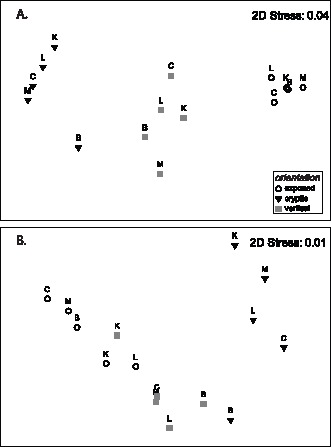
MDS ordinations of the matrices constructed from A. the benthic cover (%) of calcareous encrusters, and B. calcification rates (g m^−**2**^ y^−**1**^) for: CCA, cheilostomes, barnacles and all ‘other’ encrusters. Plots are coded for micro-habitat (exposed, cryptic, vertical) and sites identified by letter.

The one-way ANOSIM test comparing encruster communities between microhabitats gave a highly significant ANOSIM R of 0.975 (*p*<0.001). Pairwise tests between microhabitats showed significant differences: exposed ‘v’ cryptic: R  = 1 (*p*<0.01), exposed ‘v’ vertical: R  = 0.976 (*p*<0.01), cryptic ‘v’ vertical: R  = 0.936 (*p*<0.01*).* MDS ordinations (Figure 4A) gave an excellent representation of community assemblages (Stress: 0.04). There was clear separation between the community structure in the three microhabitats: exposed, cryptic and vertical and the samples displayed discrete groups. One-way SIMPER indicated that the greater abundance of cheilostomes, serpulid worms and encrusting forams in cryptic microhabitats as very important for distinguishing between the communities. Moderate and low levels of cover in the above encruster groups were observed in vertical and exposed microhabitats respectively (Figure 3).

### Encruster Calcification by Orientation

**Figure 5:**
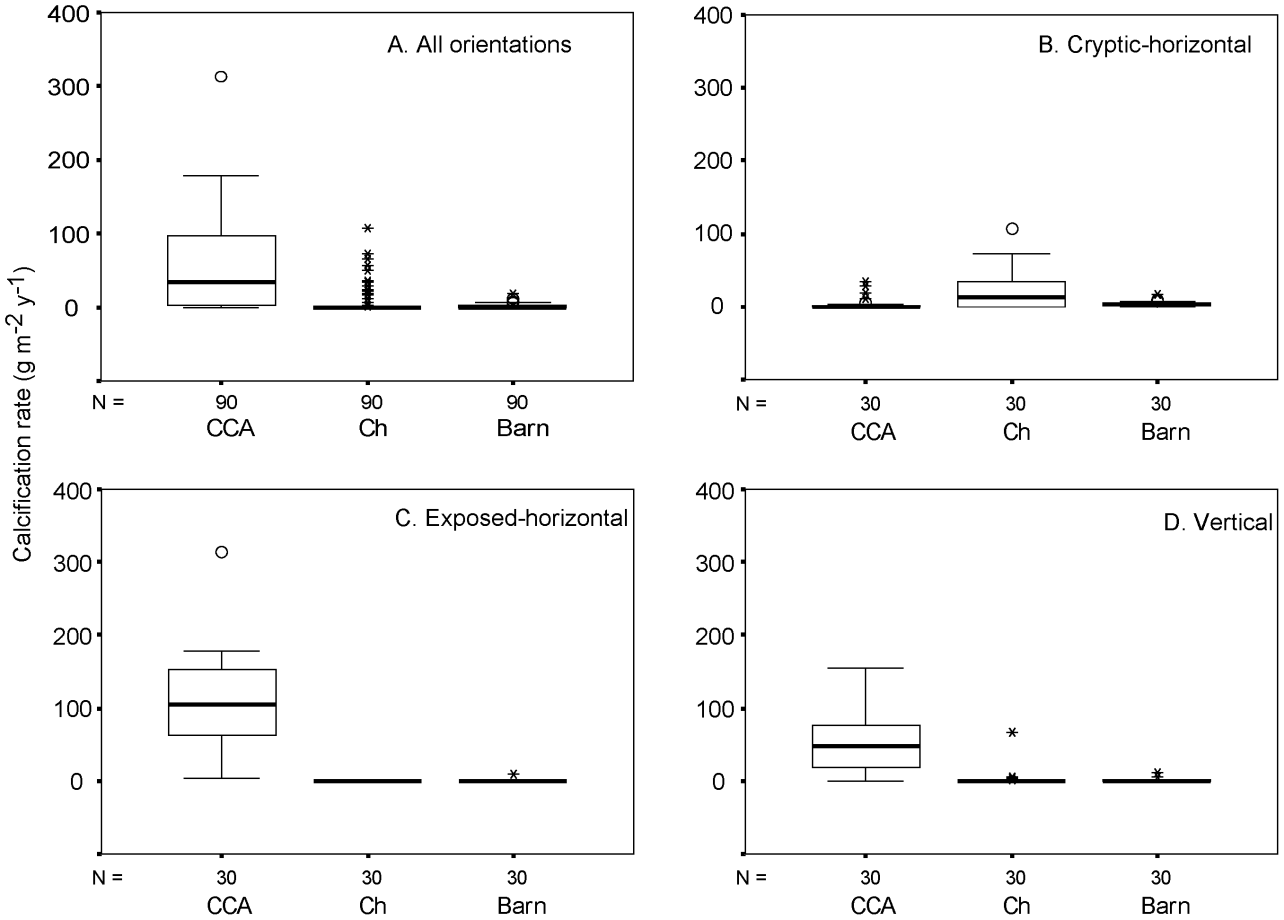
Box whisker plots detailing calcification rates (CaCO_3_ g m^−**2**^ y^−**1**^) by crustose coralline algae (CCA), cheilostome bryozoans (Ch), and barnacles (Barn) on: A. all substrates; B. cryptic substrates; C. exposed substrates; and D. vertical substrates.

**Figure 6:**
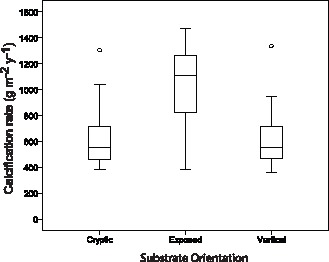
Box whisker plots detailing calcification rates (CaCO_3_ g m^−**2**^ y^−**1**^) by encrusters in different orientations (cryptic, exposed and vertical settlement plates).

Total encruster calcification rates (g m^−2^ y^−1^; Figure 5, Table 3) were significantly different between the three substrate orientations (One-way ANOVA: *F_2,87_* = 29.1, *p*<0.001) (Figure 6). A Bonferoni post-hoc comparison revealed that total calcification (mean ±SD) was similar for cryptic (619.3±216.2) and vertical (613.0±217.3) plates (*p*>0.5) but was significantly elevated (*p*<0.001) on exposed substrates (1039.3±300.9). Across all settlement plates, calcification rates (g m^−2^ y^−1^) were significantly different between CCA, cheilostomes and barnacles (Kruskal-Wallis: *H*  = 75.6, df  = 2, *p*<0.001). CCA produced the most CaCO_3_ compared to cheilostomes and barnacles (Table 3 and Figure 5). Other (non identified) encrusting organisms accounted for the majority of calcareous material (range: 356–1296 g m^−2^ y^−1^).

**Table 3:** Encruster calcification rates (g m^−2^ y^−1^) for settlement plate orientations: total (all orientations combined, n = 90), exposed (n = 30), cryptic (n = 30) and vertical (n = 30) substrates.

Organisms	Measurement	Total	cryptic	exposed	vertical
**All encrusters**	Median (interquartile range)	669.3 (540.2)	554.8 (270.1)	1108.2 (460.7)	551.9 (267.8)
	Mean (standard deviation)	757.2 (316.8)	619.3 (216.3)	1039.3 (300.9)	613.0 (217.3)
**Crustose coralline algae**	Median (interquartile range)	34.2 (96.28)	0(2.45)	105.4 (92.1)	47.9 (63.8)
	Mean (standard deviation)	55.1 (62.7)	3.8 (8.4)	105.3 (67.7)	56.3 (8.3)
**Cheilostomes**	Median (interquartile range)	0 (0)	13.5(35.2)	-	-
	Mean (standard deviation)	7.6 (19.2)	20.1 (27.0)	-	-
**Barnacles**	Median (interquartile range)	0.11 (2.86)	3.6 (2.6)	-	0.24 (1)
	Mean (standard deviation)	1.7 (3)	4.0 (3.6)	-	1.1 (2.3)

Cryptic rates of calcification (g m^−2 ^y^−1^) differed significantly between calcifying organisms (Kruskal-Wallis: *H*  = 12.2, df  = 2, *p*  = 0.002). Cheilostomes produced more skeletal carbonate framework (median ±IQR: 13.5±35.2) than CCA (median ±IQR: 0±2.45) or barnacles (median ±IQR: 3.6±2.6; Figure 5, Table 3). Other calcareous organisms accounted for 374 to 1296 g CaCO_3_ m^−2^ y^−1^. Exposed substrates displayed significant differences in calcification (Kruskal-Wallis: *H*  = 82.5, df  = 2, *p*  = 0.000) with CCA calcification rates (median ±IQR: 105.4±92.1) exceeding cheilostome (median: 0) and barnacle rates (median: 0; Figure 5, Table 3). Other calcifiers contributed from 366 to 1278 g CaCO_3_ m^−2^ y^−1^. Vertical substrates also demonstrated that the contribution to reef accretion by different encrusting organisms was significantly different (Kruskal-Wallis: *H*  = 58.9, df  = 2, *p*<0.001). Vertical calcification by CCA (median ±IQR: 47.9±63.8) was greater than cheilostomes (median: 0) and barnacles (median: 0; Figure 5, Table 3). Other encrusting organisms contributed between 357 and 1179 g CaCO_3_ m^−2^ y^−1^.

MDS ordination, coded for microhabitat, reveals excellent two dimensional representation for encruster calcification (Stress: 0.001; Figure 4B). There is little overlap between cryptic calcification and other orientations whilst exposed and vertical microhabitats display some overlap. A one-way ANOSIM test between microhabitats gave a significant ANOSIM R of 0.704 (*p*<0.001). Pairwise tests between microhabitats gave: exposed ‘v’ cryptic: R  = 0.95 (*p*<0.01), exposed ‘v’ vertical: R  = 0.564 (*p*<0.05), cryptic ‘v’ vertical: R  = 0.664 (*p*<0.01*).* SIMPER demonstrated the importance of coralline algae and ‘other’ calcifiers (e.g. serpulids, forams, bivalves) in distinguishing between the microhabitats. In all cases, these two categories contributed to >65 cumulative % of the dissimilarity between groups.

### Sediment Impacts on Encrusters

**Figure 7:**
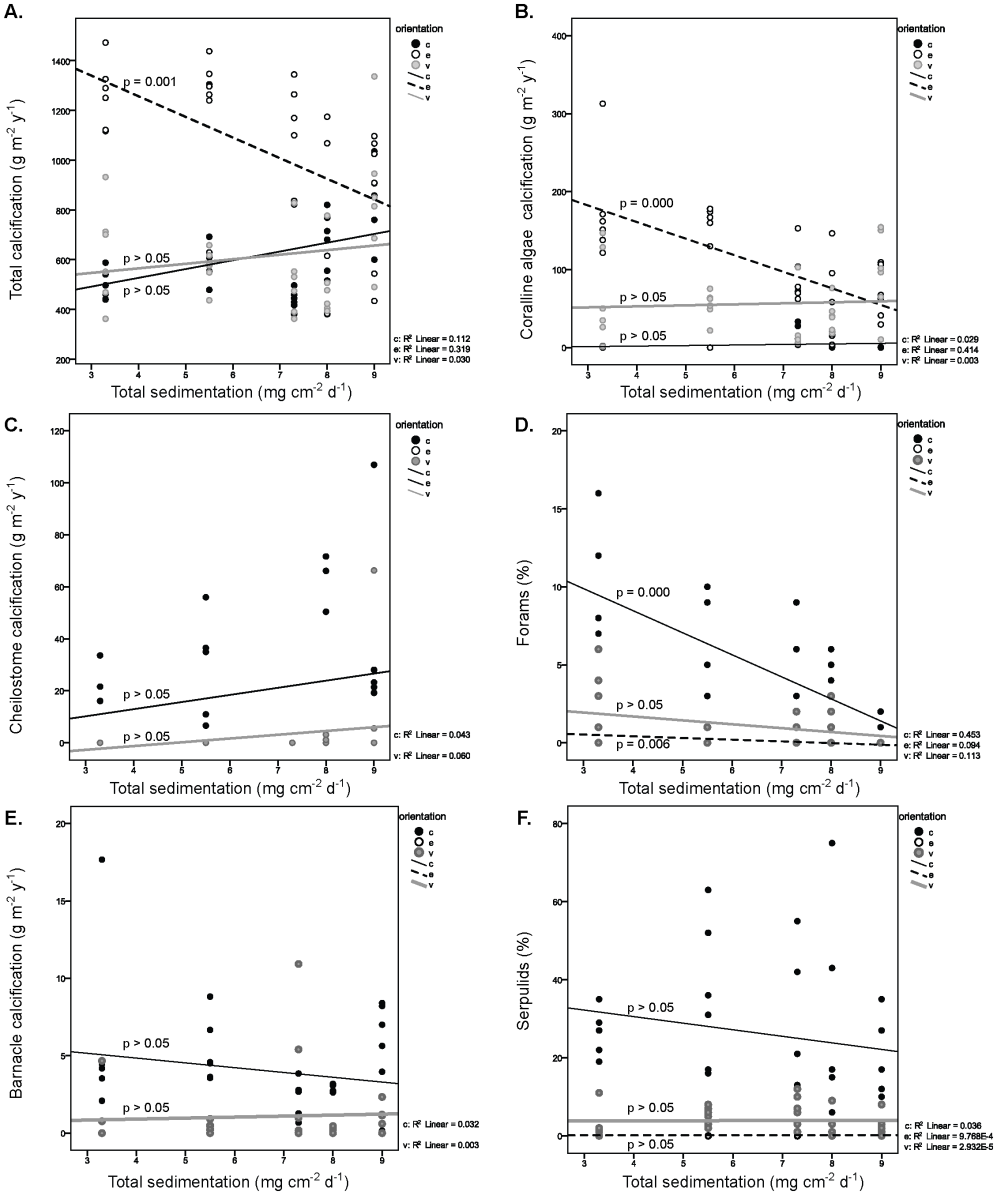
Relationships between sediment and encrusters. Correlations between total sedimentation rates and A. total calcification, B. coralline algae calcification, C. cheilostome calcification, and D. foram cover (%), E. barnacle calcification, and F. serpulid cover (%). Raw data are shown as circles, linear regression lines have been fitted.

Sedimentation rates (mg cm^−2^ d^−1^) were significantly different between the five sites (*p*<0.001; Figure 2). Total sedimentation rates were negatively correlated with rates of calcification by CCA on exposed tiles (linear regression, R^2^ = 0.41, *p*<0.001) and the cover of encrusting foraminifera on cryptic (linear regression, R^2^ = 0.45, *p*<0.001) and exposed tiles (linear regression, R^2^ = 0.09, *p*<0.01; Tables 4, 5; Figure 7 B, D). Barnacles, cheilostomes and serpulid worm communities did not show a significant relationship with sediment impacts (Figures 7C, E, F). Similarly there were no significant relationships between carbonate sedimentation rates and encruster communities (Tables 4, 5). Calcification rates for coralline algae, cheilostomes, barnacles and all groups combined are detailed by individual site and orientation in the supplementary material (Table S1).

**Table 4:** Relationship between sedimentation (total, clastic and carbonate) and encrusting foram cover and serpulids encruster cover (%).

Organism	Orientation	Total	Clastic	Carbonate
Foram	*Exposed*	−0.922*	0.052 ns	−0.700 ns
	*Cryptic*	−0.951*	−0.080 ns	−0.657 ns
	*Vertical*	−0.581 ns	−0.654 ns	−0.396 ns
Serpulid	*Exposed*	−0.279 ns	0.873 ns	−0.503 ns
	*Cryptic*	−0.594 ns	0.353 ns	−0.804 ns
	*Vertical*	0.012 ns	0.035 ns	0.182 ns

Pearsons correlation coefficient is shown. *p<0.05; ns = non-significant.

**Table 5:** Relationships between sedimentation (total, clastic and carbonate) and calcification (g m-2 y-1) by major taxa on different tile orientations.

Organism	Orientation	Total	Clastic	Carbonate
All calcifiers	*Exposed*	−0.853 ns	0.444 ns	−0.641 ns
(combined)	*Cryptic*	0.600 ns	0.397 ns	0.347 ns
	*Vertical*	0.636 ns	0.629 ns	0.347 ns
Coralline algae	*Exposed*	−0.966**	0.305 ns	−0.725 ns
	*Cryptic*	0.255 ns	−0.655 ns	0.535 ns
	*Vertical*	0.109 ns	0.437 ns	0.088 ns
Cheilostome	*Cryptic*	0.441 ns	0.435 ns	−0.048 ns
	*Vertical*	0.619 ns	0.161 ns	0.615 ns
Barnacles	*Exposed*	−0.278 ns	0.882*	−0.524 ns
	*Cryptic*	−0.299 ns	0.436 ns	−0.145 ns
	*Vertical*	0.130 ns	−0.494 ns	0.628 ns

Pearsons correlation coefficient is shown.

Note: cheilostomes were not present on exposed tiles in significant numbers.

* *p*<0.05; **p<0.01; ns = non-significant.

## Discussion

This study demonstrates the important contribution of encrusters to benthic cover and reefal carbonate accretion (total mean rate 0.8 kg m^−2^ y^−1^) when compared to estimates of global reefal carbonate production (global mean 0.9–2.7 kg m^−2^ y^−1^) [48]. The findings also provide experimental evidence that encruster calcification rates vary significantly across the reef according to microhabitat (substrate orientation: cryptic, exposed and vertical), with exposed substrates having the highest rates of calcification. However, large portions of the reefscape are composed of cryptic microhabitats (e.g. caves, cavities, overhangs and undersides of living colonies) [22] and vertical (e.g. reef wall) substrates, so it is essential for these topographic settings to be considered when assessing rates of reef accretion. Here, CCA % cover dominated exposed, and to a lesser extent, vertical fore-reef substrates. Calcification by CCA on exposed substrates was also negatively correlated with total sedimentation rates. This finding supports the view that light attenuation and the physical presence of sediment limit photosynthesis and calcification. In contrast, heterotrophs (e.g. serpulid worms and barnacles) were not negatively impacted by sediment. Calcification on cryptic substrates was dominated by ‘other’ calcifiers (e.g. serpulids, forams, bivalves), with cheilostome bryozoans, barnacles and CCA of secondary importance. These findings demonstrate that carbonate production in different reef microhabitats can be characterised by different suites of calcifying taxa which display varied rates of calcification. In addition, calcification rates can differ significantly within a taxon, depending on substrate orientation and sediment impacts. It is therefore recommended that future carbonate cycling reefscape models consider the relative contribution of different reef topographies and the extent of environmental disturbance (e.g. sediment impacts).

Organisms that dominated encruster cover (substrate area) did not necessarily produce the most skeletal carbonate in early encruster assemblages. Whilst CCA dominated benthic cover in exposed and vertical microhabitats it did not contribute the most CaCO_3_ to settlement plates. The thickness and density of the skeleton are key variables when assessing encruster calcification. As noted with corals [7], rapid linear growth does not always equate to increased rates of calcification as skeletal properties such as density and porosity are equally important. In this study, the combined carbonate input of ‘other’ calcifiers exceeded that of CCA, cheilostomes and barnacles in each experimental setting. ‘Other’ encrusters included solitary organisms such as serpulids, forams and bivalves. All display determinate growth characteristics. Consequently, growth of the calcareous skeleton is initially rapid and diminishes with age. In contrast, colonisation by colonial organisms such as CCA and cheilostomes results in rapid, lateral (linear) growth. This growth strategy gives colonial organisms a competitive advantage as they can bud, branch, overgrow and rapidly dominate available substrate. They can also overgrow the skeletons of solitary encrusters who survive unless their feeding apertures become blocked [49]. Colonial encrusters produced a thin veneer of CaCO_3_ skeleton after 12 months. The contribution of underlying organisms to CaCO_3_ production, while unquantified here, could be considerable. The density and thickness of calcareous skeletons was not assessed here. Only the surface (top most) organism was considered in these analyses. These findings highlight the important CaCO_3_ contribution of solitary organisms which may not dominate areal cover. The potential importance of underlying (overgrown) encrusters to CaCO_3_ production clearly warrants further attention.

Free space on reefs (all habitats) is typically rare, often only becoming available in patches following predation and physical disturbances. The results presented here focus on early encruster development (≤12 months) on freely available space (settlement plates). No attempt was made to account for competition for space. Longer experiments on deep (40 m) cryptic Jamaican reef environments have noted how areal cover of solitary organisms doubled in the first 7 to 14 months and then dropped sharply by 60% over the subsequent 12 months [49]. In contrast, the percentage cover of colonial organisms increased over time. It seems likely that calcification rates might also change with the age and/or size of colonial and solitary organisms. Future work should assess encruster calcification over longer timeframes and focus on mature encruster assemblages.

Results from Tobago are consistent with other observations of coralline algae dominated reefs from the Pleistocene and Holocene, for a review see [50], [51] and experimental findings in the region: Barbados [4], [25]; Curacao [52]; Jamaica [13], [49]; Lee Stocking Island and the Gulf of Mexico [53]. These studies found that calcareous organisms in high energy and/or low sediment environments were dominated by CCA and cryptic plates characterised by mixed assemblages often dominated by serpulid worms. Interestingly, total encruster calcification rates (g m^−2^ y^−1^) on exposed plates in Tobago were almost an order of magnitude greater than those reported for similarly sediment impacted (<10 mg cm^−2^ d^−1^) Jamaican fore-reef sites (mean ±SE: Tobago  = 1061.8±53.8, Jamaica  = 128.3±17.2 and 159±22), as were cryptic calcification rates (mean ±SE: Tobago  = 596.8±31.7, Jamaica  = 69.4±6 and 135.4±20) [13]. Again, these comparisons demonstrate how encruster calcification rates can vary within and among sites and the need for caution when using data sourced from other locations.

Previous studies have estimated coralline algae calcification using census-based methods to estimate percentage cover and then extrapolated growth parameter data (i.e. extension rate mm y^−1^ and skeletal density g cm^−3^) from other non-related studies. In the absence of contemporaneous, *in-situ* growth parameters, this approach is perhaps justified. However, results should be used with caution as findings are based on the assumption that such growth rate parameters are constant across all reefs and the major variable in calcification is benthic cover (%). A growing number of studies [13], [25], [54] now challenge this key assumption, documenting how encruster growth variables are influenced by environmental conditions such as light, depth, habitat, wave energy, pollution, and ocean chemistry. As global climate change (e.g. increasing frequency and magnitude of storm runoff events) continues to influence reef accretion it is hypothesised here that encrusting organisms might experience lower growth rates, weakening skeletal structure and a reduced ability to withstand biological, chemical and physical erosion. It is also likely that local stressors (e.g. catchment runoff and increasing pressure on marine natural resources) will negatively impact encruster cover and carbonate production [12], [13]. It is therefore important that we understand the implications of multiple stressors at the ecosystem level.

Census based estimates, which utilise non-site specific growth rates, of encrusting CCA carbonate production vary widely. By combining Stearn *et al.*’s [4] Caribbean skeletal density value of 1.56 g cm^−3^ with Matsuda’s [55] Pacific reef flat extension value of 1.2 mm y^−1^, carbonate production for 9 sites on the shallow shelf of the Torres Strait, Australia (15–25 m depth) were found to range from 0 to 821 g m^−2^ y^−1^ [27]. In contrast, using a skeletal density value of 1.26 g cm^−3^ [56], accretion by coralline algae onto dead *Porites* blocks in the shallows (1–2 m) in French Polynesia after a 24 month experiment ranged from 180 to 1130 g m^−2^ y^−1^ [57]. These carbonate production rates are in some cases orders of magnitude greater than site specific values reported at 10 m on reefs in Tobago (this study) and Jamaica [13]. This difference may be partly due to method and prolonged experimental exposure in French Polynesia. The Caribbean studies were conducted at greater depths on reefs subjected to periodic inputs of river runoff, where algal crusts might grow more slowly at depth due to light attenuation and limited photosynthesis [25]. Both these studies [27], [31] confirm that encruster cover and subsequent carbonate production can vary significantly at local and regional scales. Unfortunately, the use of extrapolated data from other reef locations makes fine-scale or reef habitat comparisons of calcification difficult.

Colonial bryozoans, in particular cheilostomes, are capable of overgrowing extensive areas of reef substrate [25], and records of bryozoan reef construction date back to the Ordovician [58]. Tobago’s cheilostome data suggest that they are calcifying at rates of up to 107 g m^−2^ y^−1^. Results confirm that bryozoans are still important secondary contributors to reef construction as they accrete and overgrow cryptic reef substrates. Cheilostomes typically colonised and accreted on cryptic substrates with a preference for sediment free, dimly-illuminated surfaces [13], [25]. Due to their preference for cryptic substrates, the role of encrusting bryozoans is often overlooked in reef accretion budgets. However, due to a paucity of data regarding carbonate production by tropical bryozoans their relative importance is unclear.

Filter feeding barnacles made a noticeable contribution to encruster communities on vertical and cryptic substrates in Tobago. Whilst barnacles occur in many coral reef locations, they have not formed an important component of prior reef accretion or encruster studies. As a result little is known about their contribution to the carbonate budget of tropical coral reefs. It is hypothesised here that barnacles and other heterotrophs may play a more important role in reefal carbonate production in locations that are characterised by increased sediment disturbance.

Settlement plate studies from Jamaica and Barbados note that forams and serpulid worm assemblages made a clear contribution to encruster cover at 10 m, particularly on cryptic plates which did not accumulate sediment [13], [25]. These studies noted an occasional (1–5%) contribution to vertical plates and a negligible contribution (<1%) to exposed tiles. Tobago’s cryptic assemblages were primarily characterised by serpulid worms and cheilostomes. In contrast, results from experimentally deployed bivalve shells at 15 m in Barbados and the Gulf of Mexico indicate that serpulid worms and forams dominated encruster assemblages, with cheilostomes making a negligible contribution (<1%) [53]. Calcification rates were not assessed individually for serpulids worms or forams in this study. However, the results highlight how ‘other’ organisms (including serpulids and forams) grouped together were responsible for the majority of deposited CaCO_3_. This is an area which future research should focus on.

This study might not fully represent *in-situ* calcification process on natural reefs as it relied on the use of experimental substrates deployed on coral reefs. However, there is reason to believe that it still provides useful data. Earlier comparisons of coralline algae recruitment and growth rates between natural versus experimental substrates found that results were comparable and that artificial substrates could be used to calculate carbonate production [4]. Prior census-based comparisons between natural reef substrates versus experimental settlement plates found no significant difference in encruster cover [13], [25]. This work represents an initial attempt to document carbonate production by multiple encruster groups in a framework accretion context. However, individual calcification rates were only assessed for three groups of encruster: CCA, cheilostomes and barnacles accounting for 10% of total encruster carbonate production. The encrusting organisms responsible for the remaining 90% of CaCO_3_ production need to be identified. Future studies should focus on functionally important carbonate secreting organisms (e.g. forams and serpulid worms) in a range of optimal to suboptimal reef-building environments.

Future research over longer timeframes is required to understand temporal shifts and environmental drivers of encruster calcification and the implications for reefal carbonate budgets. This study spanned 12 months and no attempt was made to account for succession or seasonal variation. The rate of occupation attained in the first 12 months to a bare substrate was rapid. However, calcification rates might increase in subsequent years as colonies mature. Early life stages are likely to be sensitive to water quality and settlement plates were deployed during Tobago’s dry season when sediment runoff (and sedimentation) was minimal. Encruster recruitment might have been less successful if substrates were deployed during the rainy season as sediment cover of available substrates can limit recruitment. Previous researchers have noted how solitary organisms often become dominated or overgrown by colonial species resulting in reduced diversity [51], [53], [59], [60]. Studies of cryptobionts in caves and under ledges also note that soft (non calcareous) encrusters have the ability to over grow and decalcify live, calcareous organisms [61]. Recent work on encrusting forams has also discovered that *Discorbis bertheloti* bioerodes carbonate substrates [51].

Ensuring the continued growth, structural integrity and topographic complexity of coral reefs is essential if they are to adapt to changing oceanic conditions. The distribution and growth of calcareous reef building organisms is heavily influenced by both abiotic factors (e.g. sediment impacts, light, and wave energy) and biotic factors (e.g. predation and competition) [25], [49], [53]. The paucity of information about these interactions in relation to encruster calcification and skeletal development makes it difficult to assess how these reef builders will handle rapid ecosystem change. Future work should focus on the impacts of increased storm and catchment runoff, elevated seawater temperatures, and changing ocean chemistry. These multiple stressors will have pronounced effects on future reef growth and the numerous reef organisms which contribute to the structural integrity and composition of reefs.

## Supporting Information

Table S1 Calcification rates (g m^−2^ y^−1^) by site (n = 5) and orientation (n = 3) for coralline algae, cheilostomes, barnacles and all groups combined.
(DOC)
