# Supplementary material for: Calcification by Reef-Building Sclerobionts
Source: PLoS One. 2013 Mar 28;8(3):e60010. doi: 10.1371/journal.pone.0060010 (PMC3610694; doi:10.1371/journal.pone.0060010)
Supplement: Table S1 — Calcification rates (g m−2 y−1) by site (n = 5) and orientation (n = 3) for coralline algae, cheilostomes, barnacles and all groups combined. (DOC) [file pone.0060010.s001.doc]

| **Kariwak Exposed** | | | | | | |
| --- | --- | --- | --- | --- | --- | --- |
| **Organism** | N | Minimum | Maximum | Mean | | Std. Deviation |
|  | Statistic | Statistic | Statistic | Statistic | Std. Error | Statistic |
| Coralline algae | 6 | 29.70 | 109.44 | 69.59 | 13.44 | 32.93 |
| Cheilostomes | 6 | .00 | .00 | .00 | .00 | .00 |
| Barnacles | 6 | .00 | .00 | .00 | .00 | .00 |
| All encrusters | 6 | 433.95 | 1096.42 | 845.70 | 116.67 | 285.78 |
| Valid N (listwise) | 6 |  |  |  |  |  |
| **Kariwak Cryptic** | | | | | | |
| **Organism** | N | Minimum | Maximum | Mean | | Std. Deviation |
|  | Statistic | Statistic | Statistic | Statistic | Std. Error | Statistic |
| Coralline algae | 6 | .00 | .60 | .18 | .12 | .29 |
| Cheilostomes | 6 | .00 | 106.95 | 33.13 | 15.28 | 37.43 |
| Barnacles | 6 | .16 | 8.40 | 5.56 | 1.28 | 3.13 |
| All encrusters | 6 | 600.00 | 1035.56 | 844.63 | 60.95 | 149.30 |
| Valid N (listwise) | 6 |  |  |  |  |  |
| **Kariwak Vertical** | | | | | | |
| **Organism** | N | Minimum | Maximum | Mean | | Std. Deviation |
|  | Statistic | Statistic | Statistic | Statistic | Std. Error | Statistic |
| Coralline algae | 6 | 10.40 | 154.44 | 96.42 | 22.13 | 54.21 |
| Cheilostomes | 6 | .00 | 66.30 | 11.97 | 10.90 | 26.71 |
| Barnacles | 6 | .00 | 2.33 | 1.07 | .32 | .77 |
| All encrusters | 6 | 490.00 | 1336.17 | 853.56 | 115.93 | 283.97 |
| Valid N (listwise) | 6 |  |  |  |  |  |
| **Buccoo Exposed** | | | | | | |
| **Organism** | N | Minimum | Maximum | Mean | | Std. Deviation |
|  | Statistic | Statistic | Statistic | Statistic | Std. Error | Statistic |
| Coralline algae | 6 | 62.41 | 153.00 | 89.94 | 13.88 | 34.01 |
| Cheilostomes | 6 | .00 | .00 | .00 | .00 | .00 |
| Barnacles | 6 | .00 | .00 | .00 | .00 | .00 |
| All encrusters | 6 | 821.73 | 1344.07 | 1089.20 | 88.98 | 217.97 |
| Valid N (listwise) | 6 |  |  |  |  |  |
| **Buccoo Cryptic** | | | | | | |
| **Organism** | N | Minimum | Maximum | Mean | | Std. Deviation |
|  | Statistic | Statistic | Statistic | Statistic | Std. Error | Statistic |
| Coralline algae | 6 | 3.44 | 33.32 | 14.37 | 5.29 | 12.97 |
| Cheilostomes | 6 | .00 | .00 | .00 | .00 | .00 |
| Barnacles | 6 | .68 | 3.85 | 2.05 | .50 | 1.23 |
| All encrusters | 6 | 381.11 | 496.54 | 438.11 | 16.01 | 39.21 |
| Valid N (listwise) | 6 |  |  |  |  |  |
| **Buccoo Vertical** | | | | | | |
| **Organism** | N | Minimum | Maximum | Mean | | Std. Deviation |
|  | Statistic | Statistic | Statistic | Statistic | Std. Error | Statistic |
| Coralline algae | 6 | 5.46 | 102.90 | 25.21 | 15.61 | 38.23 |
| Cheilostomes | 6 | .00 | .00 | .00 | .00 | .00 |
| Barnacles | 6 | .00 | 10.92 | 2.93 | 1.81 | 4.43 |
| All encrusters | 6 | 362.35 | 828.15 | 523.13 | 68.25 | 167.17 |
| Valid N (listwise) | 6 |  |  |  |  |  |
| **Mt Irvine Exposed** | | | | | | |
| **Organism** | N | Minimum | Maximum | Mean | | Std. Deviation |
|  | Statistic | Statistic | Statistic | Statistic | Std. Error | Statistic |
| Coralline algae | 6 | .00 | 178.00 | 134.87 | 27.86 | 68.25 |
| Cheilostomes | 6 | .00 | .00 | .00 | .00 | .00 |
| Barnacles | 6 | .00 | 8.82 | 1.47 | 1.47 | 3.60 |
| All encrusters | 6 | 1239.75 | 1437.53 | 1314.61 | 28.75 | 70.42 |
| Valid N (listwise) | 6 |  |  |  |  |  |
| **Mt Irvine Cryptic** | | | | | | |
| **Organism** | N | Minimum | Maximum | Mean | | Std. Deviation |
|  | Statistic | Statistic | Statistic | Statistic | Std. Error | Statistic |
| Coralline algae | 6 | .00 | .00 | .00 | .00 | .00 |
| Cheilostomes | 6 | .00 | 56.00 | 24.16 | 8.85 | 21.68 |
| Barnacles | 6 | .00 | 6.67 | 3.82 | .89 | 2.18 |
| All encrusters | 6 | 478.77 | 691.85 | 595.37 | 29.51 | 72.30 |
| Valid N (listwise) | 6 |  |  |  |  |  |
| **Mt Irvine Vertical** | | | | | | |
| **Organism** | N | Minimum | Maximum | Mean | | Std. Deviation |
|  | Statistic | Statistic | Statistic | Statistic | Std. Error | Statistic |
| Coralline algae | 6 | 22.00 | 75.53 | 54.55 | 7.49 | 18.34 |
| Cheilostomes | 6 | .00 | .00 | .00 | .00 | .00 |
| Barnacles | 6 | .00 | .91 | .35 | .14 | .35 |
| All encrusters | 6 | 436.67 | 657.90 | 570.23 | 30.80 | 75.43 |
| Valid N (listwise) | 6 |  |  |  |  |  |
| **Culloden Exposed** | | | | | | |
| **Organism** | N | Minimum | Maximum | Mean | | Std. Deviation |
|  | Statistic | Statistic | Statistic | Statistic | Std. Error | Statistic |
| Coralline algae | 6 | 121.60 | 312.84 | 176.15 | 28.26 | 69.22 |
| Cheilostomes | 6 | .00 | .00 | .00 | .00 | .00 |
| Barnacles | 6 | .00 | .00 | .00 | .00 | .00 |
| All encrusters | 6 | 1116.42 | 1471.98 | 1262.47 | 54.79 | 134.20 |
| Valid N (listwise) | 6 |  |  |  |  |  |
| **Culloden Cryptic** | | | | | | |
| **Organism** | N | Minimum | Maximum | Mean | | Std. Deviation |
|  | Statistic | Statistic | Statistic | Statistic | Std. Error | Statistic |
| Coralline algae | 6 | .00 | 2.11 | .45 | .33 | .82 |
| Cheilostomes | 6 | .00 | 33.60 | 11.88 | 5.79 | 14.19 |
| Barnacles | 6 | .00 | 17.67 | 5.32 | 2.56 | 6.27 |
| All encrusters | 6 | 439.63 | 587.78 | 494.57 | 24.37 | 59.70 |
| Valid N (listwise) | 6 |  |  |  |  |  |
| **Culloden Vertical** | | | | | | |
| **Organism** | N | Minimum | Maximum | Mean | | Std. Deviation |
|  | Statistic | Statistic | Statistic | Statistic | Std. Error | Statistic |
| Coralline algae | 6 | 1.50 | 146.94 | 64.79 | 24.11 | 59.05 |
| Cheilostomes | 6 | .00 | .00 | .00 | .00 | .00 |
| Barnacles | 6 | .00 | 4.67 | .90 | .76 | 1.87 |
| All encrusters | 6 | 362.22 | 931.85 | 621.15 | 82.95 | 203.19 |
| Valid N (listwise) | 6 |  |  |  |  |  |
| **Little Englishman's Bay Exposed** | | | | | | |
| **Organism** | N | Minimum | Maximum | Mean | | Std. Deviation |
|  | Statistic | Statistic | Statistic | Statistic | Std. Error | Statistic |
| Coralline algae | 6 | 3.75 | 146.52 | 56.15 | 22.83 | 55.92 |
| Cheilostomes | 6 | .00 | .00 | .00 | .00 | .00 |
| Barnacles | 6 | .00 | .00 | .00 | .00 | .00 |
| All encrusters | 6 | 382.47 | 1174.20 | 796.87 | 118.58 | 290.45 |
| Valid N (listwise) | 6 |  |  |  |  |  |
| **Little Englishman's Bay Cryptic** | | | | | | |
| **Organism** | N | Minimum | Maximum | Mean | | Std. Deviation |
|  | Statistic | Statistic | Statistic | Statistic | Std. Error | Statistic |
| Coralline algae | 6 | .20 | 19.72 | 3.89 | 3.18 | 7.78 |
| Cheilostomes | 6 | .00 | 71.68 | 31.37 | 14.31 | 35.06 |
| Barnacles | 6 | .00 | 3.18 | 1.94 | .62 | 1.52 |
| All encrusters | 6 | 380.74 | 820.25 | 611.23 | 64.43 | 157.81 |
| Valid N (listwise) | 6 |  |  |  |  |  |
| **Little Englishman's Bay Vertical** | | | | | | |
| **Organism** | N | Minimum | Maximum | Mean | | Std. Deviation |
|  | Statistic | Statistic | Statistic | Statistic | Std. Error | Statistic |
| Coralline algae | 6 | 18.45 | 76.44 | 40.57 | 8.43 | 20.66 |
| Cheilostomes | 6 | .00 | 3.12 | .71 | .52 | 1.26 |
| Barnacles | 6 | .00 | .45 | .12 | .08 | .19 |
| All encrusters | 6 | 393.70 | 776.91 | 497.00 | 58.76 | 143.93 |
| Valid N (listwise) | 6 |  |  |  |  |  |
